# Supplementary material for: A Novel Hypothesis on Choroideremia-Manifesting Female Carriers: Could CHM In-Frame Variants Exert a Dominant Negative Effect? A Case Report
Source: Genes (Basel). 2022 Jul 17;13(7):1268. doi: 10.3390/genes13071268 (PMC9321261; doi:10.3390/genes13071268)
Supplement: Supplementary file 1 [file genes-13-01268-s001.zip › genes-1787458-supplementary.pdf]

A

B

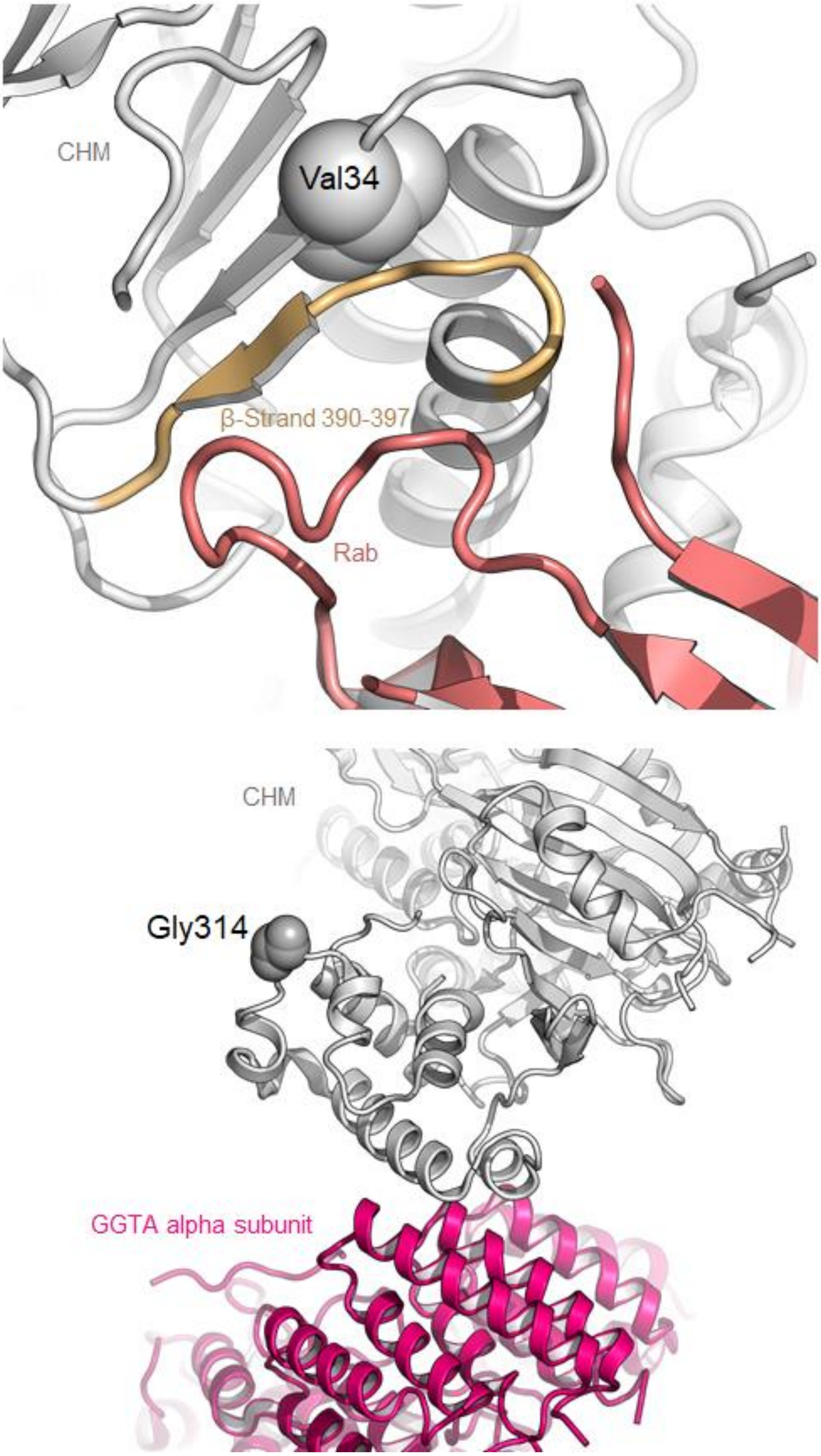

Figure S1. The p.Val34Asp and p.Gly314Arg variants mapped onto the structure of *CHM* (grey illustration). (**A**) Although it is unable to bind to Rab (pink illustrations) due to the distortion of interface residues 390–397, the p.Val34Asp mutant can still interact with GGTA (violet illustrations). (**B**) The p.Gly314Arg variant is present on the C-terminal surface of CHM but has no effect on either Rab binding nor GGTA binding.
